# *Bacillus* lipopeptide-mediated biocontrol of peanut stem rot caused by *Athelia rolfsii*

**DOI:** 10.3389/fpls.2023.1069971

**Published:** 2023-02-20

**Authors:** Virginie Korangi Alleluya, Anthony Argüelles Arias, Bianca Ribeiro, Barbara De Coninck, Catherine Helmus, Pierre Delaplace, Marc Ongena

**Affiliations:** ^1^ Microbial Processes and Interactions Laboratory, Terra Teaching and Research Center, Gembloux Agro-Bio Tech, Liège University, Gembloux, Belgium; ^2^ Chemical and Agricultural Industries, Faculty of Agricultural Sciences, University of Kinshasa, Kinshasa, Democratic Republic of Congo; ^3^ Division of Plant Biotechnics, Department of Biosystems, Faculty of Bioscience Engineering, Katholieke Universiteit (KU) Leuven, Leuven, Belgium; ^4^ Plant biology Unit, Gembloux Agro-Bio Tech, Liège University, Gembloux, Belgium

**Keywords:** *B. velezensis*, secondary metabolites, lipopeptides, *Arachis hypogaea* L., disease protection, systemic resistance

## Abstract

**Introduction:**

Peanut (*Arachis hypogaea* L.) is a widespread oilseed crop of high agricultural importance in tropical and subtropical areas. It plays a major role in the food supply in the Democratic Republic of Congo (DRC). However, one major constraint in the production of this plant is the stem rot (white mold or southern blight) disease caused by *Athelia rolfsii* which is so far controlled mainly using chemicals. Considering the harmful effect of chemical pesticides, the implementation of eco-friendly alternatives such as biological control is required for disease management in a more sustainable agriculture in the DRC as in the other developing countries concerned. *Bacillus velezensis* is among the rhizobacteria best described for its plant protective effect notably due to the production of a wide range of bioactive secondary metabolites. In this work, we wanted to evaluate the potential of *B. velezensis* strain GA1 at reducing *A. rolfsii* infection and to unravel the molecular basis of the protective effect.

**Results and discussion:**

Upon growth under the nutritional conditions dictated by peanut root exudation, the bacterium efficiently produces the three types of lipopeptides surfactin, iturin and fengycin known for their antagonistic activities against a wide range of fungal phytopathogens. By testing a range of GA1 mutants specifically repressed in the production of those metabolites, we point out an important role for iturin and another unidentified compound in the antagonistic activity against the pathogen. Biocontrol experiments performed in greenhouse further revealed the efficacy of *B. velezensis* to reduce peanut disease caused by *A. rolfsii* both *via* direct antagonism against the fungus and by stimulating systemic resistance in the host plant. As treatment with pure surfactin yielded a similar level of protection, we postulate that this lipopeptide acts as main elicitor of peanut resistance against *A. rolfsii* infection.

## 1 Introduction

Peanut (*Arachis hypogaea* L.) is one of the most widely grown legumes in the warm zones of the world cultivated for its nutritious and oil-rich pods (Arya et al, 2016). In the DRC, this crop plays a major role in the food supply and is considered as the main source of income for the producing households (Nyabyenda, 2005). However, the production of this plant is drastically impacted by various diseases including stem rot (white mold or southern blight) caused by *Athelia* (*Sclerotium*, anamorph) *rolfsii* (Curzi) Tu & Kimbrough. The stem rot disease is characterized by yellowing and wilting of the main stem, lateral branches or the whole plant. It can also attack pods in the soil leading to losses estimated at 10-40% according to the production area (Thiessen and Woodward, 2012; Iquebal et al., 2017; Bosamia et al., 2020). This disease is very difficult to eradicate notably because the fungal pathogen forms sclerotia which can remain in the soil for a considerable time and because this fungus has a wide variety of host plant species (Woodward et al., 2013; Iquebal et al., 2017; Bosamia et al., 2020). Chemical fungicides are mostly used to control this soil-borne pathogen but with limited beneficial effects due to serious problems for humans and environment (Bosamia et al., 2020; Shah, 2020). The development of more environmentally friendly alternatives is needed to manage stem rot in an efficient and sustainable way. In that context, exploiting the so-called plant-beneficial bacteria mostly isolated from the rhizosphere is considered as one of the promising alternative to unwanted chemicals (Backer et al., 2018; Mohanty et al., 2021). These rhizobacteria may promote plant growth and alleviate abiotic stresses (Kashyap et al., 2017) but in many instances, their most desired trait is to protect the host plant from infection by pathogenic microbes referred as biocontrol of plant diseases. This protective effect relies on two main mechanisms, which are direct antagonism of the pathogen and systemically expressed reinforcement of the defensive capacity of the host plant.

Isolates identified as plant-beneficial rhizobacteria with biocontrol potential belong to multiple genera including *Pseudomonas, Azospirillum, Azotobacter, Klebsiella, Burkholderia, Serratia* and *Bacillus* (Mohanty et al., 2021). Most of the *Bacillus* species reported as effective biocontrol agents belong to the so-called *Bacillus subtilis* complex and typically dwell into the soil/rhizosphere niche (Cawoy et al., 2011; Fan et al., 2017). Among these species, *B. velezensis* is considered as model for plant-associated bacilli and is the best valorized as microbial inoculant for reducing diseases caused by a wide range of pathogens with different lifecycles and modes of virulence (biotrophic, hemi-biotrophic or necrotrophic) (Fira et al., 2018; Miljaković et al., 2020; Saxena et al., 2020). *B. velezensis* also distinguishes from other species of the *B. subtilis* complex considering its richness in biosynthetic gene clusters (representing up to 13% of the whole genome) responsible for the biosynthesis of bioactive secondary metabolites (BSMs). This secondary metabolome largely contributes to the biocontrol activities of the strains and is chemically diverse, including volatiles, terpenes, non-ribosomal peptides (NRP), cyclic lipopeptides (CLPs) and polyketides (PKs), but also ribosomally-synthesized and post translationally-modified bacteriocins and lantibiotics (RiPPs) (Grubbs et al., 2017; Harwood et al., 2018). Cyclic lipopeptides are interesting amphiphilic molecules consisting of a fatty acid chain bound to a cyclic peptide ring (Harwood et al., 2018). *B. velezensis* produces three families of CLPs: surfactins, fengycins and iturins (Rabbee et al., 2019). They consist of a cyclized peptide of 7 L- or D-amino acids (iturins, surfactins) or 10 residues (fengycins) linked to an acyl chain of variable length (Caulier et al., 2019). These CLPs exhibit a strong antifungal activity but can also trigger systemic resistance in the host plant against a broad spectrum of pathogens (Romero et al., 2007; Ongena and Jacques, 2008; Pršić and Ongena, 2020)

With this work, our objective was to evaluate the understudied potential of *B. velezensis* for the biocontrol of peanut disease caused by *A. rolfsii*. To that end, we used the genetically amenable, natural rhizosphere isolate GA1 as representative of the species. Indeed, based on genome mining, this strain produces the whole panoply of bioactive secondary metabolites typically formed by members of this species (Andrić et al., 2021). Therefore, we also wanted to identify the CLPs mainly responsible for the biocontrol activity of the strain by combining different approaches including i) the determination of secondary metabolites secreted upon growth in peanut root exudates, ii) the test of various mutants repressed in CLP synthesis in *in vitro* antifungal assays and iii) the test of pure CLP *in planta* for assessing the efficiency to reduce plant infection *via* the stimulation of systemic resistance.

## 2 Materials and methods

### 2.1 Bacterial strains, media, and culture conditions

*Bacillus velezensis* strains used in this study was *B. velezensis* GA1 and its mutants (**Table 1**). Before each experiment, GA1 WT or mutants were pre-cultured as follows. Five µL of bacterial glycerol stock preserved at -80°C was streaked on Luria- Bertani (LB) medium (10 g of Tryptone/l; 5 g of yeast extract/l; 5 g of NaCl/l; 20 g of agar when needed) and incubated overnight at 30°C. One colony was used to inoculate flasks containing five times dilute LB liquid medium and agitated (160 rpm) overnight (16h) at 30°C. Pre-cultures were centrifuged at 10000 rpm for 10 minutes and bacterial cells were washed three times with LB medium. Cell concentration was evaluated (OD_600nm_) and adjusted to the desired concentration for all subsequent experiments.

**Table 1:** Bacterial strains used in this study.

Bacterial strains	Relevant characteristics	Sources
*B. velezensis* and mutants		
GA1 wt	Isolated from strawberry (*Fragaria* sp.), Italy	Touré et al., 2004
GA1 Δsrf	Surfactin deficient: itu+ fen+ srf-	Andrić et al., 2021
GA1 Δfen	Fengycin deficient: itu+ fen- srf+	Andrić et al., 2021
GA1 Δitu	Iturin deficient: itu- fen+ srf+	Andrić et al., 2021
GA1 ΔsrfΔfen	Surfactin and Fengycin deficient: itu+ fen- srf-	Lam et al., 2021
GA1 ΔituΔfen	Iturin and Fengycin deficient: itu- fen- srf+	Lam et al., 2021
GA1 ΔsrfΔitu	Surfactin and Iturin deficient: itu- fen+ srf-	Lam et al., 2021
GA1 Δsfp	Iturin, Fengycin and Surfactin deficient: itu- fen- srf-	Lab collection
GA1 ΔbacA	Bacilysin deficient: bac-	Lab collection
GA1 Δsfp-bacA	Iturin, Fengycin, Surfactin and Bacilysin deficient: itu- fen- srf- bac-	Lab collection
*Bradyrhizobium arachidis*	Isolated from peanut (*Arachis hypogaea* L.), China	Wang et al., 2013

*Bradyrhizobium arachidis* (provided by the Belgian Co-Ordinated Collections of Microorganisms “BCCM”/Bacteria Collection LMG26795) was used in this study. Before each experiments, the bacteria were streaked on Yeast Extract Mannitol Agar (YEMA) (Per liter: 10 g of Mannitol; 0.5 g of K_2_HPO_4_; 0;5 g of Sodium glutamate; 50 mg of NaCl; 1 g of yeast extract; 10 ml of Solution A (Per 100 ml:1 g of MgSO_4_.7H_2_O); 1 ml of Solution B (Per 100 ml: 5.28 g of CaCl_2_.2H_2_O); 1 ml of Solution C (Per 100 ml: 666 mg of FeCl_3_.6H_2_O); 20 g of agar). One colony was used to inoculate Yeast Extract Mannitol broth (YEM) for 3 to 7 days at 30°C and the pre-culture was washed with YEM three times. Cell concentration was evaluated (OD_600nm_) and adjusted to the desired concentration for all subsequent experiments

### 2.2 Fungal strains, media, and culture conditions

*Athelia rolfsii* (Curzi) Tu & Kimbrough (provided by the Belgian Co-Ordinated Collections of Microorganisms “BCCM”/Agro-Food and Environmental fungi MUCL 051031) was reactivated on V8 juice agar (200 ml of V8 juice; 3 g of CaCO_3_; 15 g of agar; 800 ml of distillated water; pH = 7.2). After this step, fungus was cultivated on Oatmeal agar OMA (Per liter: 60 g of oatmeal; 12.5 g of agar, pH = 7.2 ± 0.2) to produce mycelia and sclerotia at 28°C. Inoculated petri dishes were stored at 4°C or collected sclerotia were retained in peptone water (10 g/l of peptone, 5 g/l of NaCl, 1-2 g of tween 80) at the same temperature for the next experiments.

*Fusarium oxysporum*, *Aspergillus n*iger and *Rhizoctonia solani* (lab collection) were cultivated on Potato Dextrose Agar PDA (39 g/l, pH = 5.6 ± 0.2; Merck Germany) to produce mycelia. Cultivated fungi were stored at 4°C for subsequent experiments.

### 2.3 Natural peanut root exudates collection

Seeds from healthy and highly quality pods of peanut (*Arachis hypogaea* L.) cultivar JL24 (provided by National Institute for Agronomic Studies and Research (INERA) - M’vuazi in Kongo Central (DRC)) were used. Peanut seeds were surface disinfected during 2 minutes in 70% ethanol prior to another 2 minutes in 5% Sodium Hypochlorite and finally three times washed in sterile distilled water. The effect of surface sterilization was checked by spotting 50 µl of the final rinse water onto LB plates. After sterilization, seeds were deposited in a petri dish containing sterile filter paper soaked with sterile water and placed in the dark at 22°C during 5 to 7 days until the emergence of an approx. 2 cm-long radicle. Sterile seedlings were then transferred in vials container covered with aluminum foil paper to avoid light filled with 15 ml Murashige and Skoog medium (MS) for hydroponic cultivation (growth chamber conditions: 16h-day and 8h-night cycle at 22°C). After 15 days of cultivation, natural peanut root exudates (NPRE) were filtrated through a membrane (0.22 µm) and stored at 4°C for further experiments.

### 2.4 Medium mimicking peanut root exudates preparation

NPRE were collected, lyophilized, and the dry material (50 mg) was extracted using 1.2 ml of methanol by vortexing 1 minute and then sonicated at room temperature for 20 min. After centrifugation, 500 µL of the supernatant was transferred into two centrifuge tubes for measurement of sugars and acids. For sugars, 20 µL of 3 mg/ml phenyl-β-D-glucopyranoside and 20 µL of 0.1 mg/ml 3-(4-hydroxyphenyl)-propionic acid were added as internal standards. The derivatization of sugars was performed by adding 120 μL methoxyamine-hydrochloride (20 mg/ml in pyridine) for 60 min at 30°C and with 120 μL N,O-bis (trimethylsilyl) trifluoro-acetamide (BSTFA) for 120 min at 45°C, 700 rpm. For amino acids and organic acids, 20 µL of 0.1 mg/ml 3-(4-hydroxyphenyl)-propionic acid was added to the sample as the internal standard and the derivatization was performed by adding 120 µL of methoxylamin-hydrochloride (20 mg/ml in pyridine) for 90 min at 37°C, 700 rpm, followed by addition of 120µl of dimethyl-tert-butylsilyl (TBDMS) and incubated for 30 min at 60°C, 700 rpm. After centrifuging, 100 µL of the supernatant was transferred to vials for detection using TSQ Duo GC-MS/MS (Thermo Fisher Scientific).

Based on NPRE composition, medium mimicking peanut root exudates (MMPRE) was recomposed as follows: Maltose 1.09 g/l; Glucose 0.88 g/l; Arabinose 0.67 g/l; Fructose 0.28 g/l; Trehalose 0.14 g/l; Myo-inositol 0.07 g/l; Lactic acid 2.73 g/l; Succinic acid 0.13 g/l; Malonic acid 0.11 g/l; Fumaric acid 0.07 g/l; Pyruvic acid 0.03 g/l; Pyroglutamic acid 0.02 g/l; Malic acid 0.02 g/l; Citric acid 0.02 g/l; Valine 1.05 g/l; Leucine 0.57 g/l; Threonine 0.57 g/l; Isoleucine 0.42 g/l; Alanine 0.32 g/l; Phenylalanine 0.32 g/l; Tyrosine 0.15 g/l; Serine 0.14 g/l; Glycine 0.11 g/l; Asparagine 0.08 g/l; Glutamic acid 0.03 g/l. Salt and micronutrients were added with these proportions: 0.25 g/l MgSO_4_·7H_2_O; 0.34 g/l; K_2_HPO_4_; 3-morpholino-1-propanesulfonic acid MOPS 10.5 g/l; 0.25 g/l KCl, 1 g/l (NH_4_)_2_SO_4_; 1.2 mg/l Fe_2_(SO_4_)_3_; 0.4 mg/l MnSO_4_; 1.6 mg/l CuSO_4_, 4 mg/l Na_2_ MoO_4_, pH adjusted to 7. In case of assay on solid MMPRE, 20 g/l of agar was used for this preparation.

### 2.5 *Bacillus* growth and CLP production

Wild type strain GA1 and GA1-mutants were cultivated in liquid to evaluate bacterial growth and secondary metabolite production on NPRE and liquid MMPRE. Bacterial growth was evaluated by turbidity measurement (OD_600nm_) after 48 hours culture at 30°C. Relative quantification of CLP production was evaluated by UPLC-MS (Agilent 1290 Infinity II coupled with accurate mass Jet Stream ESI‐Q‐TOF 6530, Agilent) in positive mode. MS parameters were set up as follows: capillary voltage: 3.5 kV; nebulizer pressure: 35 psi; drying gas: 8 L/min; drying gas temperature: 300°C; flow rate of sheath gas: 11 L/min; sheath gas temperature: 350°C; fragmentor voltage: 175 V; skimmer voltage: 65 V; Accurate mass spectra were recorded in the range of m/z = 100-1700. Five µL of samples were injected and separated using a C18 Acquity UPLC BEH column, 2.1 × 50 mm × 1.7 μm (Waters) at a flow rate of 0.6 mL/min and a temperature of 40°C. A gradient of 0.1% formic acid (solvent A) and acetonitrile acidified with 0.1% formic acid (solvent B) was used. First, 10% B was applied during 1 min before increasing to 100% in 20 min and maintained during 3.5 min before going back to initial ratio in 0.5 min. Identification of compounds was based on retention time (in-house database) and accurate mass. Relative quantification was made according to the peak area of each metabolite.

### 2.6 Colonization of peanut roots by GA1 cells

Sterile peanut plantlets (see section 2.3) were immersed during 1 hour in 1 ml GFP tagged GA1 suspension (OD_600nm_ = 0.05) prepared as detailed in section 2.1. After this step, inoculated plantlets were transferred to vials containing 15 ml of sterile liquid Murashige and Skoog for 15 days in a growth chamber (day 16h/night 8h at 22°C). To evaluate the population, plants roots were immersed in 10 ml of peptone water solution supplemented with tween 80 (1 g/L) in which 10-15 sterile glassy beads were added to properly detach the bacterial cells from the root surface. Serial dilutions were prepared and 100 µl of each dilution were plated on solid medium LB. Plated petri dishes were then incubated at 28°C and cell emitting green fluorescence were counted using imaging system (Fusion Fx (Vilber Lourmat), AlexaFluor 488-Blue_AppStd). *Bacillus* population data were expressed to CFU/g root Dry Matter and calculated from two samples having a similar weight of root dry matter.

### 2.7 *In vitro* Antifungal experiments

Ten µL of GA1 WT or mutants’ strains (OD_600nm_ = 1) prepared as described in section 2.1 were spotted at 4.5 cm of the pathogen (*A. rolfsii*) on gelosed MMPRE. *Fusarium oxysporum*, *Aspergillus niger* and *Rhizoctonia solani* antagonism were tested in the same conditions on PDA plates. The plates were sealed with parafilm and incubated at 28°C for 6 days. The inhibition of mycelial growth was evaluated by measuring the distance between bacteria and the advancing front of the fungus. This experiment was performed using 6 independent repetitions. Impact of *Bacillus* volatiles in antagonism was performed using bipartite petri dishes. In this case, one compartment containing 10 ml of gelosed MMPRE was inoculated with 10 µl of GA1 culture (OD_600nm_ = 1) prepared as detailed in section 2.1 and sclerotia were deposited at 4.5 cm from bacteria in the other part of the petri dish. The petri dishes were doubled sealed with parafilm and incubated at 28°C for 6 days. Inhibition rate (IR) was calculated with the formula: IR (%) = (S1-S2/S1) x 100 where S1: represents the fungal area growth without bacteria, and S2 represents the fungal area growth in inoculated plates. This experiment was performed using 6 independent repetitions.

### 2.8 Biocontrol assays in greenhouse

**Figure 1:**
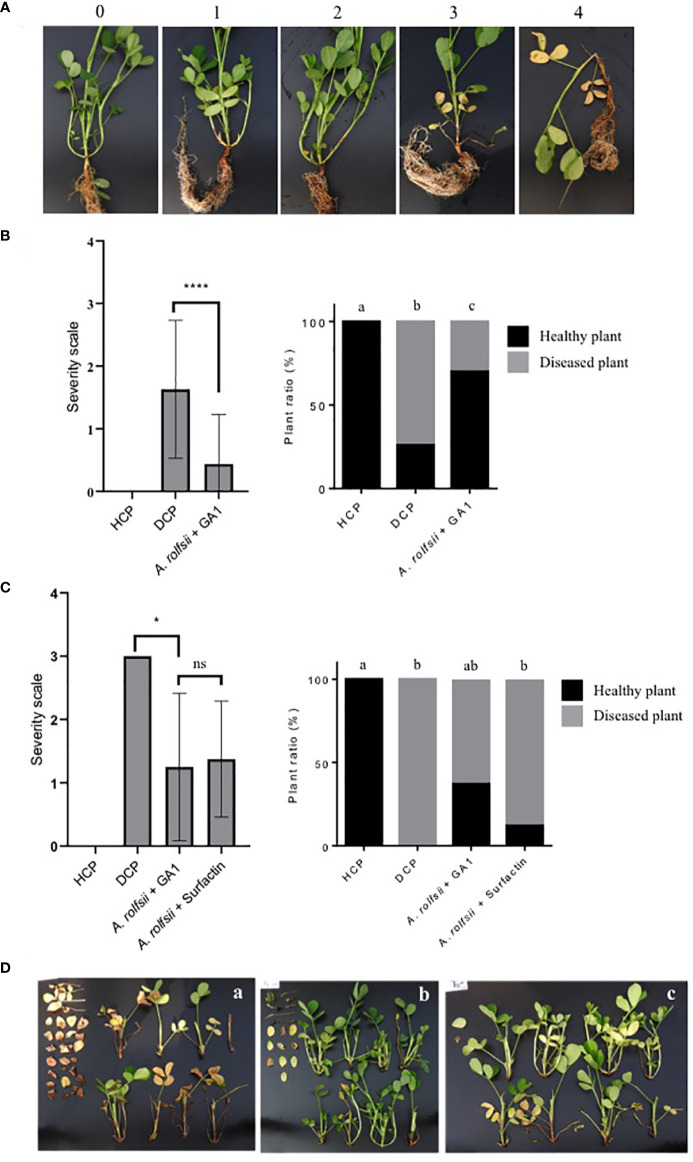
**(A)** Illustration of the severity scale used for disease rating. 0: no infection on the plant (Healthy plant); 1: mycelium present, and one branch infected; 2: mycelium present, and two branches infected; 3: mycelium present, three branches infected and beginning of leave’s yellowing; 4: three branches infected, wilting and at least 80% of leave’s yellowing. **(B)** Impact of *B. velezensis* GA1 direct antagonism against *A. rolfsii* on disease severity (left panel) and disease incidence (right graph), HCP = not infected plant, DCP = *A. rolfsii* infected plant; *A. rolfsii* + GA1 = Infected plants previously treated with *B. velezensis* GA1. Pearson’s chi-squared test followed by the pairwise tests of independence for nominal data were used to compare DI between modalities. Kruskal-Wallis’s test followed by Dunn’s multiple comparisons were used to compare DS between treatments. Means ± SD from fifty replicates of two experiments are presented. ****, p < 0.0001. Treatments with different letters are significantly different (p < 0.05). **(C)** Impact of *B. velezensis* GA1 on the induced resistance of peanuts plants against *A. rolfsii*. Disease severity (left panel) and disease incidence (right graph) are depicted; *A. rolfsii* + surfactin = Infected plants previously treated with pure surfactin (10µM/per plant). Pearson’s chi-squared test followed by the pairwise tests of independence for nominal data were used to compare DI between modalities. Kruskal-Wallis’s test followed by Dunn’s multiple comparisons were used to compare DS between treatments. Means ± SD from eight replicates of one experiment are presented. *, p<0.05 and ns (no significant difference), p > 0.05. Treatments with different letters are significantly different (p < 0.05). **(D)** Pictures showing the extent of disease symptoms as observed on several randomly collected plants for DCP **(A)**, *A. rolfsii* infected plants treated with GA1 **(B)** or infected plants treated with surfactin **(C)**. The detached leaves on the left of each image are from branches located near the collar and damaged by *A. rolfsii*.

The first experiment was performed in a greenhouse (located at Plant Clinic International/, Kinshasa - DRC). The characteristics of the soil used in this experiment were: pH: 5.35; Organic Carbon (%): 4.60; P available (ppm): 7.38; Nitrogen (%): 0.11; Potassium (ppm): 0.52; conductivity (µS/cm): 23.8. Twenty surface sterilized seeds (see section 2.3) were coated with a *Bacillus* suspension (50 ml OD_600nm_ = 1) for 10 minutes and then sown directly into plastic pots (12.5 cm diameter, 6.5 cm height) filled with 1 kg of sterilized soil (150°C, 30 hours). Before sowing one seed per pot, 10 ml of a bacterial suspension of *B. arachidis* (OD_600nm_ = 1) were deposited on the seedbed of each seed of peanut. 14 days after sowing, 3 sclerotia of *A. rolfsii* were placed on the plant collar and 10 ml of bacterial suspension of *B. arachidis* were again inoculated at the stem basis. Three days after this step, *B*. *arachidis* suspension (10 ml OD_600nm_ = 1/plant) were inoculated again by watering the stem basis and after 5 days, *B. velezensis* GA1 (50 ml OD_600nm_ = 1/plant) was applied. These two microorganisms were applied once a week for three weeks. Assessment of disease incidence (DI) and disease severity (DS) (severity scale illustrated in **Figure 1A**) were recorded one week after the last inoculation. The experiment was repeated twice with 50 replicates per treatment.

The second experiment was conducted in tropical greenhouse at TERRA/Gembloux Agro-Bio Tech in Belgium. To guarantee physical separation between GA1/surfactin and *A. rolfsii*, a system with two concentric plastic cups was used (adapted from Figueredo et al., 2017 and Rodríguez et al., 2018). Specifically, a large pot (12 x 15 x 11.5 cm) and a small pot (6 x 8.8 x 8 cm) filled with 300 g and 50 g of sterile potting soil respectively were placed on top of each other and a hole was created in the small pot to connect the two containers. Fifty seeds surface sterilized (see section 2.3) were pre-germinated in another container containing twice autoclaved potting soil for one week. Seedlings were then transplanted in the upper pot (the small) in which the roots were introduced into its hole to reach the large pot. One hour before transplanting, a suspension of *B. arachidis* (10 ml OD_600nm_ = 1/plant) was poured in each large pot. Five days after seedlings transfer, GA1 suspension (50 ml OD_600nm_ = 1/plant) or purified surfactin (10 µM/plant as indicated by the work of Rodríguez et al., 2018, purity of surfactin: 90%) were applied in the large pot. At two days after GA1/surfactin treatment, seedlings were inoculated at the stem basis (in the small pot) by adding 15 sclerotia of *A. rolfsii* and 2 plugs of agar carrying mycelia and at the same time, *B. arachidis* (10 ml OD_600nm_ = 1/plant) was applied once again. The application of *B. arachidis*, GA1 and surfactin was performed once a week for three weeks. Assessment of DI and DS (severity scale illustrated in **Figure 1A**) were recorded one week after the last inoculation. At the same time, roots nodules were counted on all plants for to evaluate the impact of GA1 inoculation on root nodulation by *B. arachidis*. The results were expressed by the number of nodules/plant. The experiment was repeated twice with 8 replicates.

### 2.9 Statistical analysis

One-way ANOVA followed by Tukey’s multiple comparisons test and graphical representatives were performed using GraphPad Prism version 8.1.0 (244) (GraphPad Software, San Diego, California USA, www.graphpad.com). One-way ANOVA (Kruskal-Wallis’s test) followed by Dunn’s multiple comparisons in GraphPad software were used specially to compare DS between treatments. The proportions of diseased plants and healthy plants (DI) after treatment by *B. velezensis* GA1 were assessed by Pearson’s chi-squared test followed by the pairwise tests of independence for nominal data. These tests were applied with the “pairwiseNominalIndependence” and the “cldList” functions of the R companion package (Mangiafico, 2016) to determine the symmetry between treatments. The significance threshold for all tests was set at 0.05%.

## 3 Results

### 3.1 *Bacillus velezensis* GA1 efficiently produces CLPs upon growth in peanut root exudates

**Figure 2:**
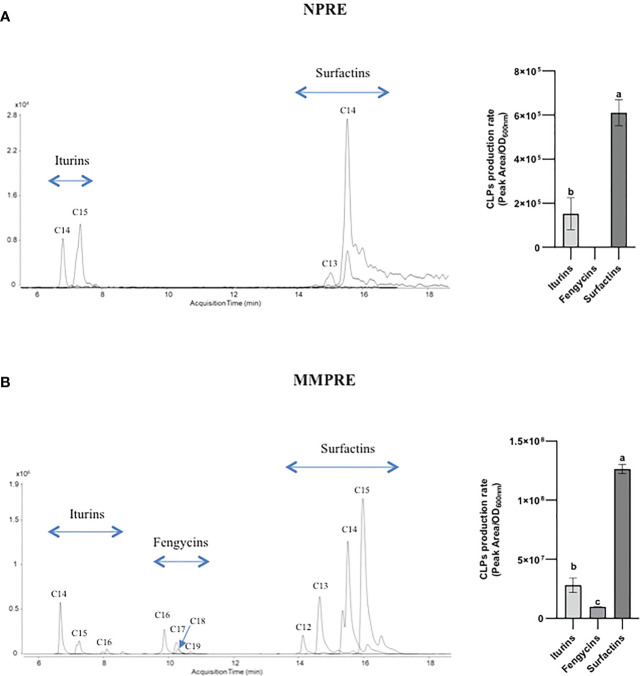
LC-MS extracted ion chromatograms (EICs) of CLPs families produced by *B velezensis* GA1 in liquid NPRE **(A)** and MMPRE **(B)** culture. The different peaks correspond to homologues of the considered family with different fatty acid chain lengths as indicated. For each medium, the relative proportions of the various CLPs have been calculated based on peak area/OD and are also represented in graphs on the right. Means ± SD from two biological replicates (with similar OD) of one experiment are shown. Treatments with different letters are significantly different (p < 0.05).

We first wanted to evaluate BSMs production by GA1 growing in root exudates collected from two-week-old peanut plants grown in hydroponics (natural peanut root exudates, NPRE). The amounts of plant chemicals utilizable by bacteria to form biomass are usually quite low in these naturally produced exudates but we observed a substantial development of GA1 with final cell density that reached an OD_600nm_ value of approx. 0.4. We also observed a good colonization potential of the strain on peanut roots as it reaches cell populations of 2.5 x 10^7^ CFU/g root (dry weight) within 4 days post inoculation. We next determined the pattern of BSMs formed by the bacterium in these conditions and UPLC-qTOFMS analysis of the corresponding cell-free crude extract revealed significant amounts of the lipopeptides iturin and surfactin but not fengycin or other non-ribosomal products potentially produced by the strain (**Figure 2A**). In terms of relative proportions determined by peak area, surfactin production rate is higher than iturin. For both CLPs, different homologues varying in the length of the fatty acid chain are co-produced (**Figure 2A**). The mass spectral data revealed the presence of iturins with fatty acid chains containing 14 and 15 carbons (C14, C15) and surfactins C13 and C14.

Using TSQ Duo GC-MS/MS, we identified 6 sugars, 8 organic acids and 11 amino acids as components in the NPRE that can be used as carbon sources by GA1. Maltose, glucose, lactic acid, valine and leucine were identified as the most prevalent compounds (**Table 2**). We created a synthetic medium based on the relative amounts of all those compounds found in NPRE and which contained 31.25% sugars, 31.25% organic acids and 37.5% amino acids in a total of 10 g/l. Higher biomass concentration was measured (OD_600nm_ = 0.73) upon growth of GA1 in this mimicking NPRE (MMPRE) together with increased amounts of CLPs compared with the production in NPRE. The relative proportions of iturins and surfactins as well as the homologue distribution within each family were similar to NPRE but detectable amounts of fengycins were observed in these conditions (**Figure 2B**). Since CLPs are quorum-sensing regulated metabolites, a higher biomass most probably led to a higher production of the three CLPs (with the same proportion), therefore allowing to detect fengycin produced above the detection limit in that case (**Figure 2B**).

**Table 2:** Quantity of soluble sugars, organic acids and amino acids detected by GC-MS in root exudates collected from 15 days-old plants of *A. hypogea* cultivar JL24 grown in hydroponics.

Compounds	Quantity (µg/mg of root exudate)	% of total
Sugars		
Maltose	0.0282	34.8
Glucose	0.0228	28.1
Arabinose	0.0174	21.5
Fructose	0.0072	8.9
Trehalose	0.0036	4.4
Myo-inositol	0.0018	2.2
Organic acids		
Lactic	0.0690	87.5
succinic	0.0032	4.1
Malonic	0.0027	3.4
Fumaric	0.0018	2.3
Pyruvic	0.0008	1.0
Pyroglutamic	0.0006	0.8
Malic	0.0004	0.5
Citric	0.0004	0.5
Amino acids		
Valine	0.0277	27.9
Leucine	0.0150	15.1
Threonine	0.0150	15.1
Isoleucine	0.0111	11.2
Alanine	0.0085	8.6
Phenylalanine	0.0084	8.5
Tyrosine	0.0041	4.1
Serine	0.0037	3.7
Glycine	0.0030	3.0
Asparagine	0.0020	2.0
Glutamic	0.0009	0.9

### 3.2 Identification of secondary metabolites involved in direct growth inhibition of *A. rolfsii*

**Figure 3:**
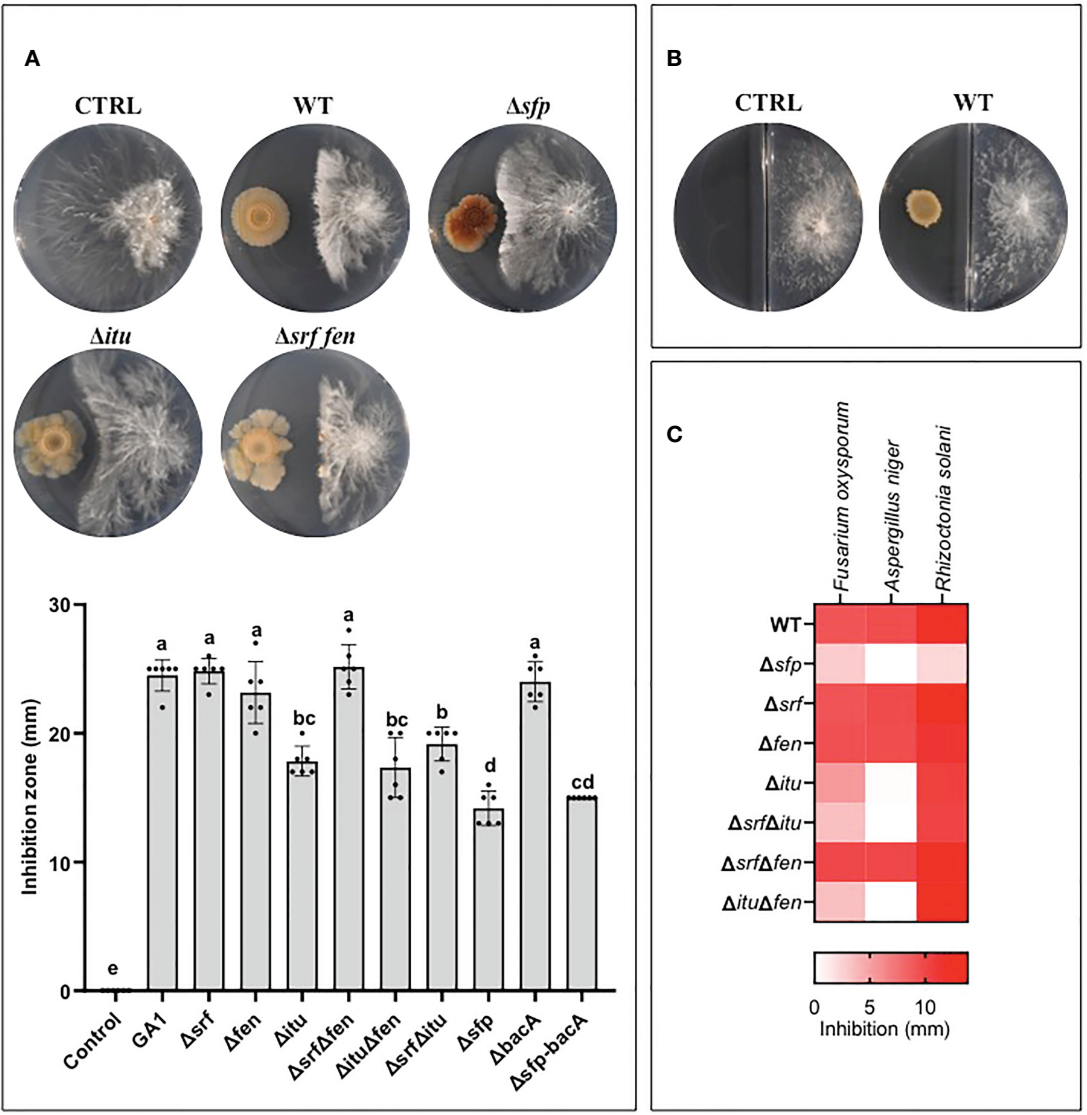
**(A)** Illustration of the antagonistic activity displayed by *B. velezensis* GA1 WT and its mutant strains impaired in production of different lipopeptides as observed on MMPRE medium against *A*. *rolfsii*. The corresponding quantitative data of inhibition by the different strains are represented in the graph below. Means ± SD from six independent repetitions of one experiment are presented. Treatments with different letters are significantly different (p<0.05). **(B)** Volatiles secreted by *B*. *velezensis* have no impact on the growth of *A*. *rolfsii* as visualized on bi-compartmented plates. **(C)** Heat map representing the level of antagonistic activity of GA1 WT and mutants against some other fungal pathogens of peanuts plants.

We next used solid MMPRE to evaluate the direct inhibition of *A. rolfsii* by GA1 in confrontation assays on plates. The wild-type strain displayed a strong inhibitory activity towards the fungus (**Figure 3A**). To determine the specific involvement of CLPs and other metabolites in bacterial inhibition, we tested a range of single and double knockout mutants of GA1. This included the Δ*sfp* derivative not able to form the 4’-phosphopantetheinyl transferase enzyme, which is essential for proper functioning of non-ribosomal peptide biosynthesis machineries responsible for the synthesis of PKs, CLPs, and bacillibactin. Data revealed that iturin plays a major role in this antifungal activity since the inhibitory effect is significantly reduced in all the mutants repressed in the synthesis of this lipopeptide (**Figure 3A**). However, another unidentified non-ribosomal compound also contributes to the fungitoxic potential of GA1 as shown by the additional loss of activity of the Δ*sfp* mutant impaired in the synthesis of all NRP- and PK-type BSMs except bacilysin. This dipeptide is non-ribosomally formed but not *via* a mechanism involving the Sfp protein and is therefore still produced in the Δ*sfp* derivative. Bacilysin was occasionally reported for its antifungal activity but is not active against *A. rolfsii* as illustrated by the fully conserved antagonistic potential of the Δ*bacA* mutant and the similar level of activity displayed by the Δ*sfp* and the double Δ*sfp*Δ*bacA* mutants (**Figure 3A**). Also, GA1 inhibition is not due to volatiles as shown by the absence of fungal growth reduction in two-compartment plates (**Figure 3B**). Iturin produced by GA1 is also partially or fully responsible for antagonism towards respectively *Fusarium oxysporum* and *Aspergillus niger* as other important peanut pathogens but not *Rhizoctonia solani* (**Figure 3C**).

### 3.3 Treatment with GA1 or purified surfactin protects peanut plants against disease caused by *A. rolfsii*

The potential of GA1 to reduce the incidence of disease caused by *A. rolfsii* on peanut plants was next tested in two different types of experiments but in both cases, the pathogen was inoculated as sclerotia on the collar of the plant. In the first type of assays, GA1 was applied both by immersing seeds in a cell suspension just before sowing and by drenching with GA1 cell suspension at the stem basis of two weeks-old plants. Disease rating was done based on macroscopic observation of symptoms as illustrated in **Figure 1A**. The biocontrol potential of GA1 was evaluated approx. three weeks after infection based on reduction in disease incidence and disease severity compared with control plants infected by *A. rolfsii* but not treated with the bacterium (**Figure 1B**). Application of GA1 provided a significant 60% reduction of the disease based on incidence (X^2^ = 61.166, df = 2, p<0.0001) and severity (p<0.0001) (**Figure 1B**). As there is no spatial separation between the two microbes in this experimental set-up, disease control is obviously due to direct antagonism of the pathogen. In a second type of assay, we wanted to evaluate the ability of GA1 to protect the host plant *via* the induction of systemic resistance. We used a system with two concentric pots to guarantee spatial separation between GA1 and the fungal pathogen. Based on disease severity, a significant protective effect (p<0.0001) was observed by comparing *A rolfsii*-infected plants (disease control) and infected plants previously treated with GA1 (**Figures 1C, D**). Interestingly, treatment with the purified lipopeptide surfactin (soil drenching with a 10μM solution/per plant) also provided a similar level of disease severity reduction. Considering disease incidence, we observed a reduction in the number of infected plants of 12.5% and 37.5% upon treatment with pure surfactin and with GA1 cells respectively (X^2^ = 20.267, df = 3, p-value = 0.0001495) (**Figures 1C, D**).

### 3.4 GA1 alters root nodulation by *B. arachidis*

**Figure 4:**
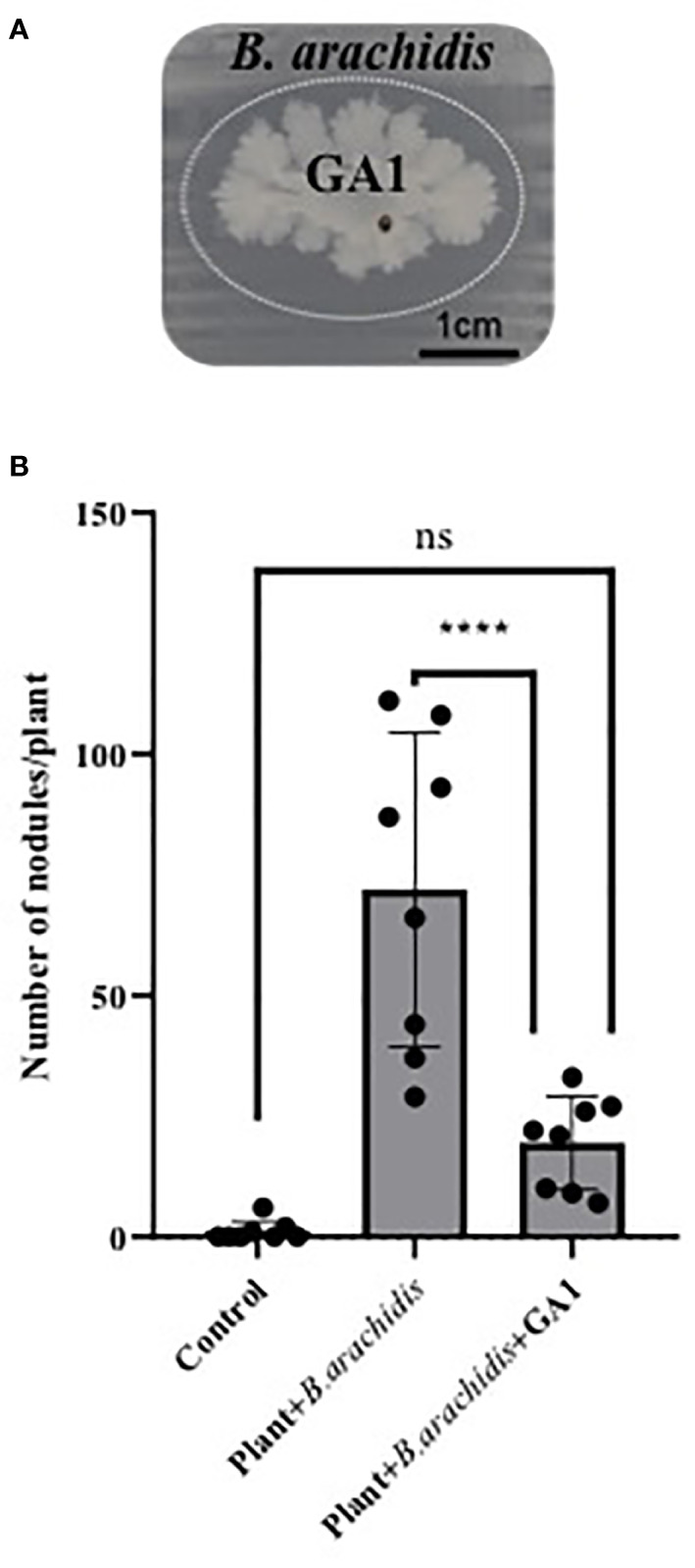
**(A)** Antagonistic effect of *Bacillus velezensis* GA1 (growing colony at the center) against *Bradyrhizobium arachidis* (growing as thin layer of cells over the whole surface) on Yeast Extract Mannitol Agar. It is visualized by the inhibition zone delimited by a dotted elliptic line. **(B)** Impact of *Bacillus* inoculation on *B. arachidis* nodulation on roots evaluated based on the number of nodules in each treatment; Plant: non-inoculated plants; Plant + *B. arachidis*: *A. rolfsii* infected peanut plants inoculated with *B. arachidis* Plant + *B. arachidis* + GA1: *A. rolfsii* infected peanut plants inoculated with *B. arachidis* and *B. velezensis* GA1.Values are the mean ± SD from eight replicates of one experiment. ****,p < 0.0001 and ns (no significant difference), p > 0.05.

Peanut belongs to the Fabaceae family which naturally establishes symbiosis with rhizobia helping the plant to acquire nitrogen from atmospheric N_2_ (Peix et al., 2015). We therefore wanted to test the compatibility of *B. velezensis* with the development of these important symbionts. Upon *in vitro* confrontation on Yeast Extract Mannitol Agar, which allows consistent growth of the symbiont, GA1 displays some slight inhibitory activity against *B. arachidis* selected as model symbiotic rhizobium (**Figure 4A**). We used the same plants as described above (see section 2.8) to assess whether GA1 may impact the ability of *B. arachidis* to form nodules on roots of infected plants. Data indicated that the nodulation rate on roots is significantly reduced in presence of GA1 (**Figure 4B**) but not abolished indicating that *B. velezensis* does not fully exclude *Bradyrhizobium* from the rhizosphere niche.

## 4 Discussion

Microbial physiology and production of secondary metabolites is often studied in laboratory conditions upon growth in artificial rich media which do not reflect the nutritional conditions encountered in natural settings. Plant-associated and beneficial rhizobacteria such as *B. velezensis* primarily use root exuded compounds as carbon and nitrogen sources to multiply and efficiently establish on host tissues and this may strongly impact their behaviour and the synthesis of bioactive small-size metabolites (Fan et al., 2012; Nihorimbere et al., 2012; Qiu et al., 2014; Ogran et al., 2019). It is why we performed an in-depth analysis of the chemicals released by roots of *A. hypogaea* plants, allowing to provide a first detailed profiling of exuded products for this species. We show that *B. velezensis* efficiently form cyclic lipopeptides of the iturin and surfactin families by growing on these exudates as sole nutrient source. In addition, the bacterium readily uses the naturally exuded compounds as source of nutrients and display high root colonization potential of peanut roots. Collectively, these data strongly suggest that a consistent production of CLPs also occur *in planta*.

Iturins are well known for their toxicity against a broad range of phytopathogenic fungi and oomycetes (Gong et al., 2014; Luna-Bulbarela et al., 2018; Jin et al., 2020; Wang et al., 2020). Here, we show that this type of CLP is the main antifungal compound secreted by GA1 responsible for direct inhibition of the peanut pathogen *A. rolfsii*. This indicates that iturin production also plays a key role for the protection observed *in planta* when GA1 and the fungal pathogen develop in contact or in close vicinity. Nevertheless, our data show that another non-ribosomal compound also contributes to the antagonistic activity of the GA1 strain. This rules out a possible role for bacteriocins and terpenes which can also be formed by GA1 based on the presence of corresponding biosynthetic gene clusters (BGCs) in the genome (Arguelles-Arias et al., 2009; Caulier et al., 2019). The polyketides bacillaene, difficidin and macrolactin are other non-ribosomal sfp-dependent secondary metabolites known for their antimicrobial activity. However, possible involvement of those compounds in the GA1 antagonistic potential is not obvious since i) they were not detected in cell-free extracts upon growth on MMPRE and because these BSMs are almost exclusively described as anti-bacterial agents not active on fungi (Müller et al., 2014; Wu et al., 2015; Im et al., 2020; Anckaert et al., 2021). Other candidate molecules are linear lipopeptides displaying antifungal properties as recently reported in *B. subtilis* (Chakraborty et al., 2020) but no BGC encoding such kind of molecule has been identified in the genome of the strain and, except those corresponding to iturins, surfactins and fengycins, no other molecular feature displaying fragmentation pattern typical of (lipo)peptidic compounds were detected in the GA1 cell-free extracts upon UPLC-qTOF-MS analysis (not shown). Identification of this possibly new metabolite certainly deserves further investigation as it may be important in the broad context of natural product discovery but also more specifically as additional weapon driving the biocontrol performance of the *B. velezensis* species.

Surfactin is also readily produced by *B. velezensis* developing in the specific nutritional context established by peanut root exudation. In general, this lipopeptide does not display remarkable antimicrobial activity when tested at the biologically relevant low micromolar concentrations (Andric et al, 2020; Anckaert et al., 2021). According to our data, surfactin is also not involved in the antibiosis activity of GA1 toward *A. rolfsii*. However, as recently reported (Rodríguez et al., 2018), we show that this CLP retains a huge potential to stimulate systemic resistance in peanut when tested as root treatment on plants infected by the pathogen on aerial parts. Such a plant resistance-triggering activity of surfactin has been previously reported in various plants and is associated with the priming of specific defense pathways that remain to be deciphered in the case of peanut (Ongena et al., 2007; García-Gutiérrez et al., 2013; Chowdhury et al., 2015; Debois et al., 2015; Pršić and Ongena, 2020). Our data strongly suggest that disease reduction observed upon treatment with GA1 in a set-up where it remains spatially separated from *A. rolfsii* is mediated *via* a surfactin-triggered boost of peanut plant immunity.

Our results show the high potential of *B. velezensis* strain GA1 to protect peanut from disease caused by *A. rolfsii*, an economically important pathogen causing stem rot. Other *Bacillus* isolates belonging to various species of the *subtilis* subgroup have also been reported as strong antagonists of this phytopathogen *in vitro* (Ahmad et al., 2019; Chen et al., 2020; Xu et al., 2020; Kumari et al., 2021). However, studies demonstrating biocontrol of this disease under greenhouse or field conditions are rare (Tonelli et al., 2011; Figueredo et al., 2017) and the mechanisms underlying this protective effect are poorly documented even if some involvement of lipopeptides and volatiles were evoked based on the potential of the tested strains to form these compounds *in vitro* (Munjal et al., 2016; Agisha et al., 2019; Chen et al., 2019). With this work, we thus provide new insights into the role played by CLPs in direct antibiosis and induction of systemic resistance in the *Arachis hypogaea/A. rolfsii* pathosystem.

Importantly, a successful biocontrol agent must meet a number of criteria, including being ecologically compatible with other plant-beneficial bacteria such as rhizobia in leguminous plants (Mohanty et al., 2021). Upon *in vitro* confrontation on solid medium, we observed some inhibition of *Bradyrhizobium* by GA1 but to a quite limited extent compared with what is commonly observed when *B. velezensis* develops its antagonistic potential against phytopathogenic fungi or bacteria. Still this reflects that some exoproducts formed by *B. velezensis* are toxic for *Bradyrhizobium*. The antagonistic effect of GA1 was more clearly observed. *In planta*, this negative effect was more marked and root-treatment with GA1 led to a significant reduction in the density of rhizobial nodules. It may be due to niche exclusion since *B. velezensis*, as good colonizer, may outcompete the rhizobium for root invasion but it may also rely on an antagonistic interaction involving secondary metabolites. *In vitro* confrontation tests were performed on a medium very different from the MMPRE medium mimicking root exudates and we assume that the natural nutritional conditions may be favorable for a higher production of compounds responsible for rhizobium inhibition in the peanut rhizosphere compared to *in vitro* on plates. That said, inoculation of *B. velezensis* did not fully impaired *Bradyrhizobium* development *in planta* indicating that the two beneficial bacteria may co-exist in accordance with previous studies reporting such compatibility between soil bacilli and various rhizobial isolates on peanut (Abd-Allah and El-Didamony, 2007; Figueredo et al., 2014) and soybean plants (*Glycine max* L.) also belonging to the Fabaceae family (Sibponkrung et al., 2020).

A better understanding of the molecular processes driving such multitrophic interactions between the host plant and mutualistic, symbiotic and pathogenic microbes is necessary, but our work provide new insights into the mechanisms used by *B. velezensis* to protect peanut against an important fungal disease. This further support the potential of the species as powerful biocontrol agent keeping in mind that rational management of its mode of application is required to avoid negative impact on natural symbiosis, which is crucial for the ecology and health of such kind of crop.

## Data availability statement

The raw data supporting the conclusions of this article will be made available by the authors, without undue reservation.

## Author contributions

VKA performed most of the microbiology and greenhouse experiments with the technical support of CH and advisory help of PD. AAA performed metabolomics using UPLC-MS and data analysis. BR and BC performed the analysis for root exudate composition. VKA, AAA, and MO mainly wrote the manuscript. MO supervised the study. All authors contributed to the article and approved the submitted version.
